# Retrograde Amnesia – A Question of Disturbed Calcium Levels?

**DOI:** 10.3389/fncel.2021.746198

**Published:** 2021-12-17

**Authors:** Dirk Montag

**Affiliations:** Neurogenetics Laboratory, Leibniz Institute for Neurobiology, Magdeburg, Germany

**Keywords:** retrograde amnesia, neuroplastin, PMCA, associative memory, memory loss, dementia, post-traumatic stress disorder (PTSD)

## Abstract

Retrograde amnesia is the inability to remember events or information. The successful acquisition and memory of information is required before retrograde amnesia may occur. Often, the trigger for retrograde amnesia is a traumatic event. Loss of memories may be caused in two ways: either by loss/erasure of the memory itself or by the inability to access the memory, which is still present. In general, memories and learning are associated with a positive connotation although the extinction of unpleasant experiences and memories of traumatic events may be highly welcome. In contrast to the many experimental models addressing learning deficits caused by anterograde amnesia, the incapability to acquire new information, retrograde amnesia could so far only be investigated sporadically in human patients and in a limited number of model systems. Apart from models and diseases in which neurodegeneration or dementia like Alzheimer’s disease result in loss of memory, retrograde amnesia can be elicited by various drugs of which alcohol is the most prominent one and exemplifies the non-specific effects and the variable duration. External or internal impacts like traumatic brain injury, stroke, or electroconvulsive treatments may similarly result in variable degrees of retrograde amnesia. In this review, I will discuss a new genetic approach to induce retrograde amnesia in a mouse model and raise the hypothesis that retrograde amnesia is caused by altered intracellular calcium homeostasis. Recently, we observed that neuronal loss of neuroplastin resulted in retrograde amnesia specifically for associative memories. Neuroplastin is tightly linked to the expression of the main Ca^2+^ extruding pumps, the plasma membrane calcium ATPases (PMCAs). Therefore, neuronal loss of neuroplastin may block the retrieval and storage of associative memories by interference with Ca^2+^ signaling cascades. The possibility to elicit retrograde amnesia in a controlled manner allows to investigate the underlying mechanisms and may provide a deeper understanding of the molecular and circuit processes of memory.

## Introduction

Retrograde amnesia is the backward loss of memories and is distinguished from forward anterograde amnesia, which is the inability to acquire new memories. Retrograde amnesia may be caused by a single specific traumatic event or it may accompany brain disorders or malfunctions. Traumatic events can be a direct impact by injury, viral or bacterial infections, malnutrition (Korsakoff’s syndrome), or psychoactive drugs and also psychogenic experiences (for review, see Squire and Alvarez, 1995). Memories can be affected by retrograde amnesia generally but also distinct types of memories such as associative memories may be selectively compromised. Usually, “normal” forgetting (Nørby, 2018), such as forgetting information without significance, is not referred to as retrograde amnesia although the mechanisms underlying forgetting and retrograde amnesia may not be distinct.

Forgetting relevant information and memory loss commonly have negative connotations. However, in cases of traumatic experiences or negatively connotated “bad” memories, forgetting may be highly welcome. Indeed, forgetting “negative” experiences and emphasizing “good” memories may help coping with life and generating optimism (Nørby, 2018). Furthermore, an increasing number of patients suffers from post-traumatic stress disorder (according to the United States National Center for PTSD 7 or 8 out of 100 people will experience PTSD at some point in their live^1^). In particular, associative memories underlie post-traumatic stress disorder because the traumatic experience is linked to the memory of other stimuli/items/signals that later on may become triggers for the painful/traumatic recall.

In contrast to learning or memory acquisition studies, the literature on experimentally induced retrograde amnesia is very scarce. Besides studies of patients suffering from retrograde amnesia which often lead to rather anecdotal knowledge, experimental access to retrograde amnesia is very limited. Experimentally, retrograde amnesia can be induced by some psychoactive drugs from which alcohol is the most prominent (for review: Lee et al., 2009). However, the specificity and mechanisms of memory loss induced by psychoactive drugs are not precisely understood.

Recently, we observed in a genetically engineered mouse model, that the induced loss of neuroplastin resulted in retrograde amnesia of associative memories (Bhattacharya et al., 2017). The very surprising finding that the specific loss of associative memories can be elicited by reduction of the expression of the protein neuroplastin from neurons will be reviewed here in detail.

## Neuroplastin, a Cell Recognition Molecule of the Immunoglobulin Superfamily Acts Also as a Subunit of Plasma Membrane Calcium Atpases

Neuroplastin is a type I glycoprotein belonging to the immunoglobulin superfamily (Langnaese et al., 1997). Polymorphisms in the regulatory region of the human *NPTN* gene correlate with cortical thickness and intellectual abilities in adolescents (Desrivières et al., 2014) and were detected in individuals suffering from schizophrenia (Saito et al., 2007). Recently, the *NPTN* gene has been associated with heart rate (Choy et al., 2018) and lung cancer (Sumardika et al., 2019). Furthermore, neuroplastin is essential for hearing (Lin et al., 2021a) and plays important roles in the immune system (Korthals et al., 2017). The adhesive and synaptic functions of neuroplastin and its role in neuronal plasticity were reviewed previously (Owczarek and Berezin, 2012; Beesley P. et al., 2014; Beesley P. W. et al., 2014). Our recent review summarizes the role of neuroplastin and its binding partners in molecular pathways underlying neuropsychiatric and neurodegenerative diseases such as schizophrenia, depression, or Alzheimer’s disease (Lin et al., 2021b).

Phylogenetically, an anchestor ortholog of the neuroplastin gene originated before divergence of vertebrates and invertebrates. In Drosophila, the single ortholog is designated basigin, however, it shares similar degrees of homology with the mammalian neuroplastin and basigin genes. A first gene duplication event gave rise to embigin and a second to neuroplastin and basigin yielding three paralogs in vertebrates. In mammals, the three paralogs neuroplastin, basigin (CD147, EMMPRIN and other names), and embigin comprise the small basigin gene family (Yates et al., 2020).

**FIGURE 1:**
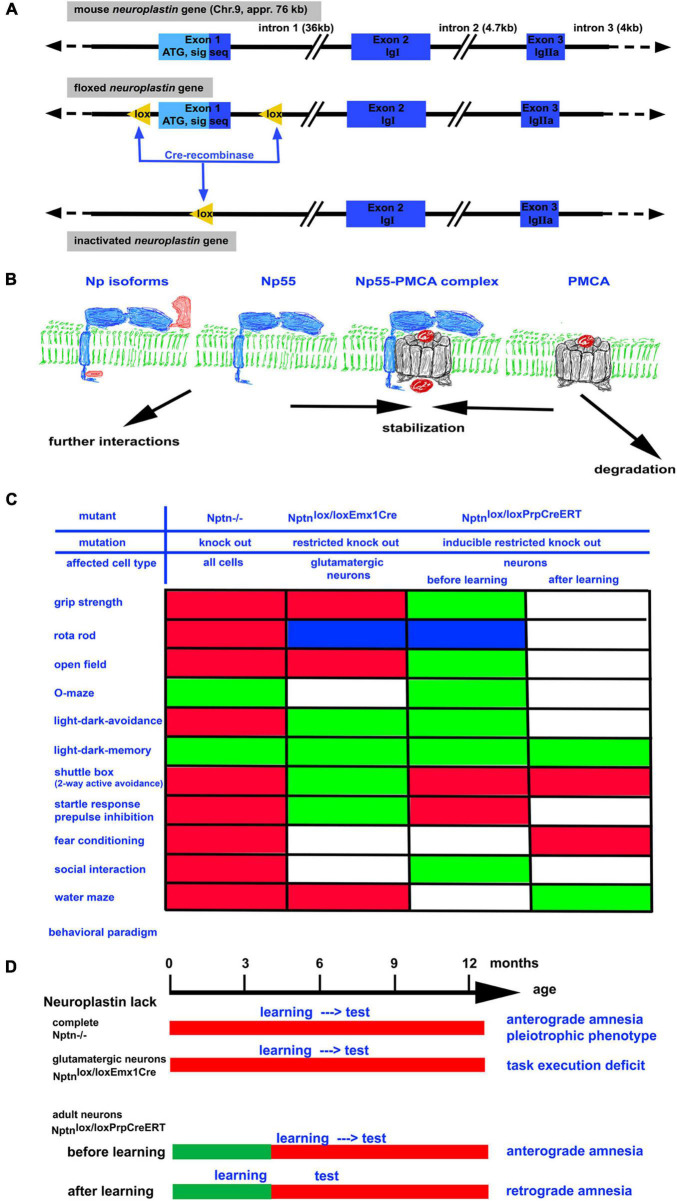
**(A)** The neuroplastin gene is localized on chromosome 9 in the mouse. It has a size of approximately 76 kb. By homologous recombination in embryonic stem cells, a floxed neuroplastin allele was generated in which the first exon encoding the start codon ATG and the signal sequence is surrounded by lox recognition sequences for the Cre recombinase (Bhattacharya et al., 2017). Cre recombinase activity leads to deletion of the first exon generating a neuroplastin null allele. By use of different Cre recombinases (constitutive, conditional, and/or inducible) the neuroplastin allele can be inactivated *in vivo* in all cells, in specific cell types, and/or in specific cell types at a chosen time point. **(B)** Neuroplastin (blue) is expressed as 55 kD isoform containing 2 Ig domains or as the brain specific 65 kD isoform containing 3 Ig domains. Both isoforms may contain the intracellular DDEP amino acid motif resulting from alternative splicing. Complex formation of neuroplastin with PMCA results in efficient Ca^2+^ extrusion from the cell. In the absence of neuroplastin, the amount of PMCAs rapidly decreases. **(C)** Summary of abnormalities detected by various behavioral paradigms in neuroplastin mutants. Green boxes: performance like control; red boxes: performance negatively affected by the mutation; blue boxes: performance improved by the mutation; white boxes: not determined. **(D)** The time schedule for the assessment of learning and memory using different neuroplastin mutants. Note that retrograde amnesia can be induced when training occurs before inactivation of the neuroplastin gene.

The mammalian neuroplastin gene encodes four isoforms derived by alternative splicing of the exons encoding the first immunoglobulin (Ig 1) domain or a small peptide (DDEP) in the cytoplasmic part (Figure 1). The neuroplastin isoforms are designated according to the apparent molecular weight of the glycosylated proteins with Np65 referring to isoforms containing all three Ig-domains and Np55 referring to isoforms containing only Ig-domains 2 and 3. All isoforms have a single pass transmembrane domain and a short cytoplasmic domain of 34 or 38 amino acids depending on the insertion of the four amino acids DDEP (Langnaese et al., 1997; Kreutz et al., 2001).

The neuroplastin gene is widely expressed in many organs but not in all cells. For example, neuroplastin is expressed in the brain by neurons but not by glia (Langnaese et al., 1997). The Np65 isoform is restricted to brain neurons, whereas Np55 is expressed in many tissues (Hill et al., 1988; Langnaese et al., 1997; Smalla et al., 2000).

A variety of interaction partners for neuroplastin has been identified. In particular, interactions with the fibroblast growth factor (FGF) receptor (Owczarek et al., 2010), gamma-aminobutyric acid type A (GABAA) receptors (Sarto-Jackson et al., 2012; Herrera-Molina et al., 2014), S100A8/A9, basigin (Sakaguchi et al., 2016; Sumardika et al., 2019), mesencephalic astrocyte-derived neurotrophic factor (MANF; Yagi et al., 2020), tumor necrosis factor receptor-associated factor 6 (TRAF6; Vemula et al., 2020), AMPA Receptor (Jiang et al., 2021), and with itself undergoing homophilic binding (Langnaese et al., 1997; Smalla et al., 2000; Owczarek et al., 2011) were reported. Furthermore, monocarboxylic acid transporter 2 (MCT-2, SLC16A7) and XK-related protein 8 (Xkr8) may require neuroplastin as chaperone (Wilson et al., 2013; Suzuki et al., 2016).

Neuroplastin supports the expression of plasma membrane Ca^2+^ ATPases (PMCAs; Bhattacharya et al., 2017; Herrera-Molina et al., 2017) by engaging in tight contact forming functional complexes with PMCAs (Herrera-Molina et al., 2017; Korthals et al., 2017; Schmidt et al., 2017; Gong et al., 2018). In the absence of neuroplastin, PMCA levels are reduced resulting in elevated intracellular Ca^2+^ levels and prolonged decay time to reach resting Ca^2+^ levels after stimulation (Herrera-Molina et al., 2017; Korthals et al., 2017; Schmidt et al., 2017).

The functions of neuroplastin were also investigated using targeted mouse mutants with specific loss of only the Np65 isoform (Amuti et al., 2016; Li et al., 2019), complete loss of neuroplastin (*Nptn^TM 1⋅2Mtg^*, Bhattacharya et al., 2017), conditional loss in glutamatergic neurons (*Nptn^lox/loxEmx1 Cre^*, Herrera-Molina et al., 2017), or inducible conditional neuron-specific mutants (*Nptn^lox/loxPr–CreERT^*, Bhattacharya et al., 2017). These studies proved that neuroplastin is important for multiple pathways and functions. The loss of neuroplastin affects cellular functions as Ca^2+^ homeostasis, long-term-potentiation, synapse formation, and more (Bhattacharya et al., 2017; Korthals et al., 2017) resulting ultimately in pronounced deficits including male infertility, depression-like behavior, and learning and memory deficits in *Nptn* mutants (Bhattacharya et al., 2017). Np65 appears to serve particular functions related to cognitive functions and the synaptic balance (Amuti et al., 2016; Li et al., 2019). Furthermore, particular mutations in the neuroplastin gene or its ablation result in deafness (Carrott et al., 2016; Zeng et al., 2016; Lin et al., 2021a).

## The Neuroplastin Mouse Model: A New Genetic Approach to Study Retrograde Amnesia

The generation of mouse mutants carrying a floxed neuroplastin gene (Figure 1; Bhattacharya et al., 2017) opened the possibilities to generate mouse strains that do not express neuroplastin at all (knock-out, KO) and sublines in which neuroplastin can be inactivated conditionally e.g., restricted to certain tissues or cell-types. With these mice, the function of neuroplastin in particular neuron types or for particular processes can be investigated. Importantly, the effect of Nptn ablation in neurons after successful acquisition of a task could be investigated (Bhattacharya et al., 2017).

Mouse mutants lacking neuroplastin completely (KO) display multiple general deficits such as a smaller body size, a reduced life expectancy, hormonal dysregulation, distinct behavioral abnormalities (Figure 1), hearing deficits, and the inability of neuroplastin-deficient male mice to reproduce (Bhattacharya et al., 2017; Lin et al., 2021a). These pleiotrophic deficits indicate a more general role of neuroplastin that is required for the maintenance of many systems. In addition, there are also deficits specifically affecting learning and memory. In particular, associative learning is strongly compromised in neuroplastin-deficient mice (Bhattacharya et al., 2017). When trained in a two-way active avoidance paradigm (shuttle box), which used light as the signal to step into the other compartment in order to escape a foot-shock, mice lacking neuroplastin were unable to successfully acquire this task whereas control mice showed about 85% correct responses. Similarly, when loss of neuroplastin was induced specifically in neurons (*Nptn^lox/loxPrCreERT^*) in adult mice, the animals were not able to learn the shuttle box paradigm. Neuronal neuroplastin was required for the acquisition of this associative memory task even when a normal development had taken place. The largest amount (appr. 95%) of neuronal neuroplastin is expressed by glutamatergic neurons. Accordingly, mutants which lack neuroplastin only in glutamatergic neurons display distinct behavioral abnormalities (Herrera-Molina et al., 2017; Figure 1). Particularly task execution behaviors that may be related to problems with goal directed behavior are affected in these mutants. Surprisingly, mutants lacking neuroplastin in glutamatergic neurons showed slightly improved associative learning (Herrera-Molina et al., 2017). These results argue that neuroplastin expressed by non-glutamatergic neurons such as gaba-ergic neurons is very important for associative learning. In support of this interpretation, disinhibition of cortical gaba-ergic interneurons plays a decisive role in fear associated learning (for review, Letzkus et al., 2015). In summary, the experiments where neuroplastin was lacking in all neurons of the brain during the training of the associative tasks clearly revealed the necessity of neuroplastin during acquisition.

The role of neuroplastin for associative memories was revealed in mice that were trained in the presence of neuroplastin and then loss of neuroplastin was induced (Bhattacharya et al., 2017). *Nptn^lox/loxPrCreERT^* mice were trained in the two-way active avoidance paradigm to high performance (≥80% correct conditioned responses) before inducible inactivation of neuroplastin specifically in neurons. After induction of the inducible cre-recombinase with tamoxifen to inactivate the neuroplastin gene, neuroplastin protein present during the training was slowly degraded. At 2 months after induction, only low levels of neuroplastin remained detectable and the mice were unable to perform the previously acquired shuttle box task. Thus, these mice displayed retrograde amnesia. Further retraining of these mice did not result in performance at high levels. The retrograde amnesia was also associated with an inability to relearn. Similar results were obtained using a fear conditioning paradigm showing that retrograde amnesia did not depend on the specific paradigm and that in particular associative memories were lost. In control experiments with the identical animals, it was shown that the retrograde amnesia was specific for associative memories but that spatial memories (Morris water maze paradigm) or memories for the environment (light-dark box paradigm) were not affected by loss of neuroplastin. Therefore, the memory system that initially worked perfectly and stored the information lost specifically the recall/retrieval/access of the associative memories upon neuroplastin loss after learning.

## The Calcium Hypothesis: A Potential Mechanism Underlying Retrograde Amnesia Caused by Neuroplastin Loss

Formation of associative memories requires the integration of information from different modalities: sensory input (e.g., reception of light stimulus, foot shock, environment -escape possibility) and emotional value (e.g., punishment foot shock, internal reward escape). This makes evident that different brain areas including cortex, hippocampus, amygdala, and more are involved and cooperate during learning and acquisition. However, it is not clear where the integration and storage are located and how the information is encoded. For associative memory formation, a critical role of disinhibition by cortical GABAergic interneurons has been proposed (for review, Letzkus et al., 2015). The comparison of neuroplastin ablation only in glutamatergic neurons (Herrera-Molina et al., 2017) versus ablation in all neurons (Bhattacharya et al., 2017) points to GABAergic interneurons as decisive components for this acquisition process. For the execution of the proper response, the stored information needs then to be retrieved and funneled into motor control circuits.

As mentioned above, neuroplastin engages in numerous interactions with other molecules (for review: Lin et al., 2021b) and disturbance of these interactions might be involved in retrograde amnesia. However, the recent identification of a decisive role of neuroplastin for Ca^2+^ extrusion *via* PMCAs suggests an attractive integrating explanation. Interestingly, previous studies using the ß-blocker propranolol revealed the importance of Ca^2+^ homeostasis for the retrieval of negative emotional experiences and that propranolol ameliorated post-traumatic stress disorder (Menzles, 2012; for review, Lonergan et al., 2013).

It is tempting to speculate that the underlying cause for retrograde amnesia after neuroplastin reduction is an altered Ca^2+^-level. We and others have shown that neuroplastin interacts with PMCAs and that the reduction of neuroplastin is accompanied with reduction of PMCA proteins (Bhattacharya et al., 2017; Herrera-Molina et al., 2017; Korthals et al., 2017; Schmidt et al., 2017) but not affecting PMCA mRNA levels (Herrera-Molina et al., 2017). The scheme in Figure 1 summarizes the hypothetical molecular events, where the interaction of neuroplastin with PMCA leads to stabilization of PMCA. The neuroplastin-PMCA complexes may engage further intracellular interactions sequestering these complexes and tethering these to the places where Ca^2+^ extrusion to the extracellular space is required. In these complexes PMCAs would be withdrawn from degradation. In the absence or after loss of neuroplastin, the amount of PMCAs is reduced (Bhattacharya et al., 2017). The normal PMCA mRNA levels after neuroplastin loss (Herrera-Molina et al., 2017) suggest that newly synthesized PMCAs are quickly degraded if they are not stabilized e.g., by neuroplastin. In neuronal cell cultures, the lack of neuroplastin slows down Ca^2+^ extrusion after stimulation and the intracellular Ca^2+^ levels remain elevated (Herrera-Molina et al., 2017). Therefore, it is feasible, that loss of neuroplastin allows PMCA degradation resulting in inappropriately high Ca^2+^ levels which interfere with signal transmission. This may cause changing network activities that may finally impair retrieval or result in loss of the memory trace.

In the future, it should be examined whether neuroplastin expressed by gabaergic interneurons is the decisive component for memories and whether retrieval or storage or both are affected by neuroplastin loss.

## Author Contributions

DM wrote the manuscript and designed the figure.

## Conflict of Interest

The author declares that the research was conducted in the absence of any commercial or financial relationships that could be construed as a potential conflict of interest.

## Publisher’s Note

All claims expressed in this article are solely those of the authors and do not necessarily represent those of their affiliated organizations, or those of the publisher, the editors and the reviewers. Any product that may be evaluated in this article, or claim that may be made by its manufacturer, is not guaranteed or endorsed by the publisher.

## Footnotes

1
https://www.ptsd.va.gov/understand/common/common_adults.asp
